# Clinical characteristics and visual outcomes of animal-induced ocular injuries: a prospective multicenter study in Iran

**DOI:** 10.3389/fmed.2024.1462252

**Published:** 2024-09-30

**Authors:** Mohammad Mohammadi, Alireza Attar, Golnoush Mahmoudinezhad, Armita Shahesmaeilinejad, Dagny Zhu, Brian Fowler, Yeganeh Farsi, Mohammad Shirvani, Mohsen Gohari

**Affiliations:** ^1^School of Medicine, Shahid Sadoughi University of Medical Sciences, Yazd, Iran; ^2^Poostchi Ophthalmology Research Center, Department of Ophthalmology, School of Medicine, Shiraz University of Medical Sciences, Shiraz, Iran; ^3^Hamilton Glaucoma Center, Shiley Eye Institute, Viterbi Family Department of Ophthalmology, University of California San Diego, La Jolla, CA, United States; ^4^HIV/STI Surveillance Research Center, and WHO Collaborating Center for HIV Surveillance, Institute for Futures Studies in Health, Kerman University of Medical Sciences, Kerman, Iran; ^5^NVISION Eye Centers, Rowland Heights, California, CA, United States; ^6^Department of Ophthalmology, The University of Tennessee Health Science Center, Hamilton Eye Institute, Memphis, TN, United States; ^7^School of Medicine, Shahid Beheshti University of Medical Sciences, Tehran, Iran; ^8^Geriatric Ophthalmology Research Center, Shahid Sadoughi University of Medical Sciences, Yazd, Iran

**Keywords:** ocular injury, eye, animal, trauma, animal bite

## Abstract

**Background:**

Animal-induced ocular injuries represent an under-documented health problem that may pose significant visual complications. This study aimed to investigate the clinical characteristics and outcomes of ocular injuries caused by animals.

**Methods:**

This multicenter prospective study enrolled patients with a history of animal-induced ocular injuries presenting to the ophthalmology departments of two tertiary hospitals over a one-year period. All participants underwent comprehensive assessments by ophthalmologists, and the following data were collected: demographic information, animal species involved, injury details, pre- and post-treatment visual data, management strategies, and follow-up outcomes.

**Results:**

Seventy-two patients (62.5% male) were included in the study. Insects were the most common species, causing 37.5% of ocular injuries. The type of animal involved was significantly associated with injury patterns (*p* < 0.0001), visual impairments at presentation (*p* < 0.05), and need for surgery (*p* < 0.001). Insects predominantly caused periorbital soft tissue injuries; birds primarily affected the anterior segment; dogs and cats mainly led to adnexal injuries; and equines often involved both anterior and posterior segments. Equine-related injuries resulted in the most severe visual impairments, requiring surgical interventions in all cases. At the same time, the majority of cases involved with other animal species experienced no visual impairment. The number of patients without visual impairment increased from 46 cases (63.9%) at the time of presentation to 58 (80.6%) at discharge after treatment. At follow-up, eight patients (11.1%) experienced complications, including traumatic cataract (*n* = 6, 8.3%), endophthalmitis (*n* = 3, 4.2%), corneal scarring (*n* = 1, 1.4%), and retinal detachment (*n* = 1, 1.4%). Rooster pecking was the leading cause of complications, including endophthalmitis and traumatic cataract.

**Conclusion:**

This study revealed that depending on the type of animal causing the injury, animal-induced ocular injuries present with diverse characteristics, requiring appropriate treatment approaches and potentially resulting in different outcomes. These findings may promote public awareness and improve preventive strategies and clinical guidelines development.

## 1 Introduction

Ocular injuries caused by animals are an under-documented health problem that may be severe and lead to permanent visual loss and cosmetic disfigurements (1). Though the World Health Organization has reported animal bites as a significant health concern affecting millions of people annually, the prevalence of animal-induced ocular injuries remains unclear (2).

These injuries are more common among the pediatric age group and may result from the interaction of humans and domestic or non-domestic animals (1).

Depending on the type of animal and its behavior, animal-induced ocular injuries may potentially range from superficial abrasions and lacerations to more severe conditions such as intraocular hemorrhages, retinal detachments, orbital bone fractures, and globe ruptures (3). Moreover, there is a risk of infectious complications such as endophthalmitis as well as tetanus and rabies due to the transmission of microorganisms in animal-induced ocular injuries (3). Moreover, in some cases, such as injuries caused by arthropods, the toxic effects of the injected venom may lead to significant complications, such as optic neuropathy and branch retinal vein occlusion (4, 5).

Therefore, a comprehensive ophthalmological examination, along with an early and appropriate therapeutic strategy with close follow-up, is crucial to achieving visual improvement and preventing subsequent complications (5). The management of animal-induced ocular injuries consists of infection treatment, surgical repair of damaged tissue, proper rabies and tetanus prophylaxis, and informing public health officials (6). Nevertheless, despite prompt and appropriate management, 14% of cases experience complications, and 8.7% of patients require revision surgery (7).

Due to the uncommon nature of animal-induced ocular injuries, the majority of the literature on these injuries has been limited to case reports and series (8–11), and there is no specific guideline for the optimal management of affected individuals. Improving understanding the effect of these injuries may optimize patient care and promote public awareness about the importance of preventive measures when interacting with animals. This prospective multicenter study aimed to address clinical features, management strategies, and visual outcomes of animal-induced ocular injuries.

## 2 Methods

This multicenter prospective study enrolled patients with a history of animal-induced ocular injuries presenting to the ophthalmology departments of the Khalili Hospital, Shiraz, Iran, and Shahid Sadoughi Hospital, Yazd, Iran, during a one-year period (from September 17, 2022 to October 7, 2023). The present study was conducted according to the Declaration of Helsinki, and the study protocol was approved by the Ethics Committee of Shahid Sadoughi University of Medical Sciences, Yazd, Iran (ethics code: IR.SSU.MEDICINE.REC.1402.287). Written informed consent was obtained from all the patients included in the study.

Participants included cases with ocular injuries caused by animal interactions. Individuals with pre-existing ocular conditions or injuries that could confound the assessment of the animal-induced ocular injury, as well as those who did not return for follow-up visits, were excluded.

For all of the cases included in the study, a comprehensive history, including demographic information of cases, type of animal species causing injury, details of the injury and ocular examination results before and after treatment, information on the management of the injury, and follow-up outcomes, were obtained by the ophthalmologist. Data was collected using structured questionnaires.

Medical records of included patients were reviewed, and the following parameters were investigated in detail including sex, age, the season of injury, species of animal involved, eye laterality, details of the injury, visual acuity (VA) at the time of presentation and discharge, management approaches, follow-up dates, visual outcomes, and complications at the last follow-up visit.

The ocular trauma score (OTS) for each injury was calculated based on the method proposed by Kuhn et al. (12). The OTS employs a numerical scale ranging from 1 to 5 to evaluate the severity of ocular injuries and predict the prognosis at a 6-month follow-up period (12). A score of 1 indicates the most severe injury with the poorest prognosis, while a score of 5 represents mild injury with the most favorable prognosis (12).

Additionally, VA data were classified into seven categories according to the International Classification of Diseases version 11: category 0 (10/10 > VA ≥ 5/10, representing no visual impairment), category 1 (5/10 > VA ≥ 3/10, representing mild visual impairment), category 2 (3/10 > VA ≥ 1/10, representing moderate visual impairment), category 3 (1/10 > VA ≥ 1/20, representing severe visual impairment), category 4 (1/20 > VA ≥ 1/50, representing blindness), category 5 (1/50 > VA ≥ light perception, representing blindness), and category 6 (no light perception, representing blindness). Categories 4 through 6 are defined as blindness (13).

Descriptive statistics was used to characterize the study population. For categorical variables, data is presented in frequencies and percentages. For continuous variables, median and interquartile range (IQR) were used. The Fisher exact test was used to compare the relative frequency of injuries, VA data, and the need for surgery across different types of animals causing injuries. Data were analyzed using SPSS software, version 23. A *p*-value less than 0.05 was considered significant.

## 3 Results

### 3.1 Demographic of patients

A total of 72 patients (male–female ratio of 1.7:1) with a diagnosis of animal-induced ocular injuries were included in this study. The median age of patients was 25 years, with an IQR of 32.25 years. Table 1 summarizes the distribution of patients’ age groups and the seasonal pattern of injuries.

**Table 1:** Demographic characteristics of patients.

Variable		Frequency (%)
Age (years)	7 <	12 (16.7)
	7–12	9 (12.5)
	13–20	10 (13.9)
	21–30	10 (13.9)
	31–40	12 (16.7)
	41–50	5 (6.9)
	51–60	5 (6.9)
	> 60	9 (12.5)
Gender	Female	27 (37.5)
	Male	45 (62.5)
Season of injury	Spring	20 (27.8)
	Summer	26 (36.1)
	Fall	18 (25.0)
	Winter	8 (11.1)

### 3.2 Animals

Insects (*N* = 27, 37.5%) were the most common cause of injuries, followed by birds (*N* = 16, 22.2%), cows, sheep, and goats group (*N* = 15, 19.7%), dogs and cats (*N* = 8, 11.1%), equines (*N* = 5, 6.9%), and arachnids (*N* = 1, 1.4%). The detailed distribution of animal species involved in ocular injuries is summarized in Table 2.

**Table 2:** Classification of animal species causing ocular injuries.

Animal species		Frequency (%)
Insects	Insects of unknown species	15 (20.8)
	Bee	11 (15.3)
	Ant	1 (1.4)
Arachnids	Scorpion	1 (1.4)
Birds	Rooster/Hen	8 (11.1)
	Minah bird	8 (11.1)
Dogs and Cats	Dog	5 (6.9)
	Cat	3 (4.2)
Cows/Sheep/Goats	Cow	11 (15.2)
	Sheep	1 (1.3)
	Goat	3 (4.2)
Equines	Horse	4 (5.5)
	Donkey	1 (1.4)
Total		72 (100)

All (*N* = 8, 100%) dogs and cats causing ocular injuries were family pets and familiar to their victims.

### 3.3 Presentations and injury characteristics

Table 3 shows the distribution of injuries and their involvement patterns. All patients experienced unilateral ocular injuries following animal interactions.

**Table 3:** Distribution of injuries and their involvement patterns.

Variable		Frequency (%)
Compromised eye	Right eye	41 (56.9)
	Left eye	31 (43.1)
	Both eyes	0 (0)
Anterior segment injuries	Gross hyphema	7 (9.7)
	Conjunctival laceration	6 (8.3)
	Sub conjunctival hemorrhage	6 (8.3)
	Full thickness corneal laceration	6 (8.3)
	Corneal epithelial defect	4 (5.6)
	Microhyphema	3 (4.2)
	Corneal ulcer	4 (5.6)
	Full thickness sceleral laceration	2 (2.8)
	Partial thickness corneal laceration	2 (2.8)
	Partial thickness scleral laceration	1 (1.4)
	Intra corneal foreign body	1 (1.4)
	Sub conjunctival foreign body	1 (1.4)
	Iridodialysis	1 (1.4)
	Globe rapture	1 (1.4)
	Traumatic cataract	1 (1.4)
Posterior segment injuries	Retinal tear	2 (2.8)
	Vitreous hemorrhage	2 (2.8)
	Retinal detachment	2 (2.8)
Adenexal injuries	Upper lid laceration	8 (11.1)
	Canalicular laceration	2 (2.8)
	Lid margin laceration	1 (1.4)
	Retrobulbar hemorrhage	1 (1.4)
	Three wall fracture	1 (1.4)
	Lower lid laceration	1 (1.4)
Periorbital soft tissue injuries	Preseptal cellulitis	15 (20.8)
	Periorbital echymosis	4 (5.6)
	Periorbital edema	3 (4.2)
Involvement pattern	Anterior segment only	33 (45.8)
	Periorbital soft tissue only	18 (25)
	Adnexa only	10 (13.9)
	Anterior segment + Posterior segment	4 (5.6)
	Anterior segment + Periorbital soft tissue	4 (5.6)
	Anterior segment + Adnexa	2 (2.8)
	Posterior segment only	1 (1.4)

**Figure 1:**
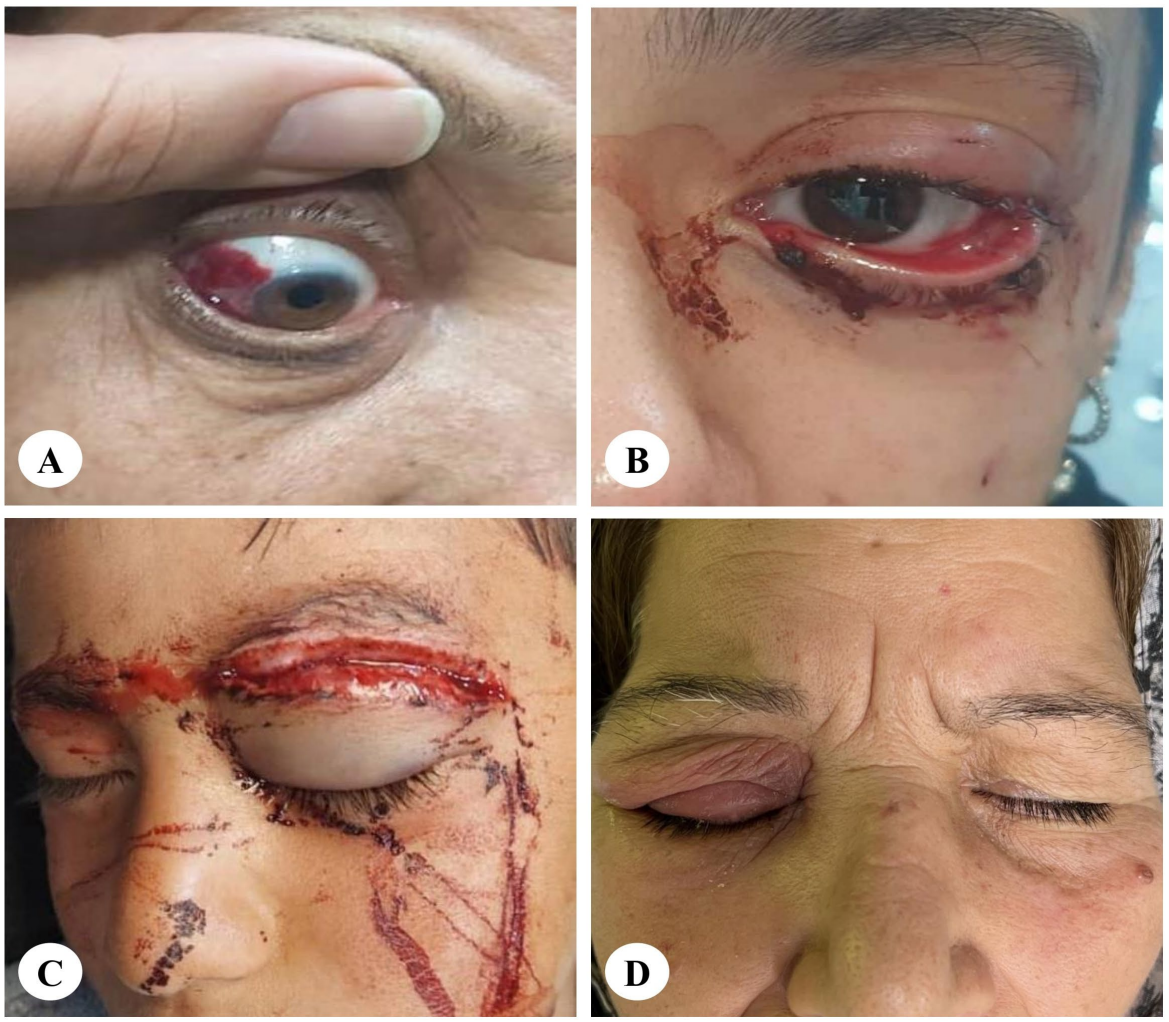
Several ocular injuries caused by animals. **(A)** Subconjunctival hemorrhage caused by a hen peck. **(B)** Canalicular laceration caused by a dog bite. **(C)** Upper lid laceration caused by a horse kick. **(D)** Preseptal cellulitis caused by an insect bite.

The injuries were categorized into four main groups: anterior segment, posterior segment, adnexal, and periorbital soft tissue injuries. The most common injuries included preseptal cellulitis in 20.8% (*N* = 15) of cases, upper lid laceration in 11.1% (*N* = 8), gross hyphema in 9.7% (*N* = 7), and conjunctival laceration, subconjunctival hemorrhage, and full-thickness corneal laceration, each present in 8.3% (*N* = 6) of cases. Figure 1 shows several ocular injuries caused by animals.

The involvement of the anterior segment only was the most common pattern of injuries, occurring in 45.8% (*N* = 33) of cases (Table 3).

**Figure 2:**
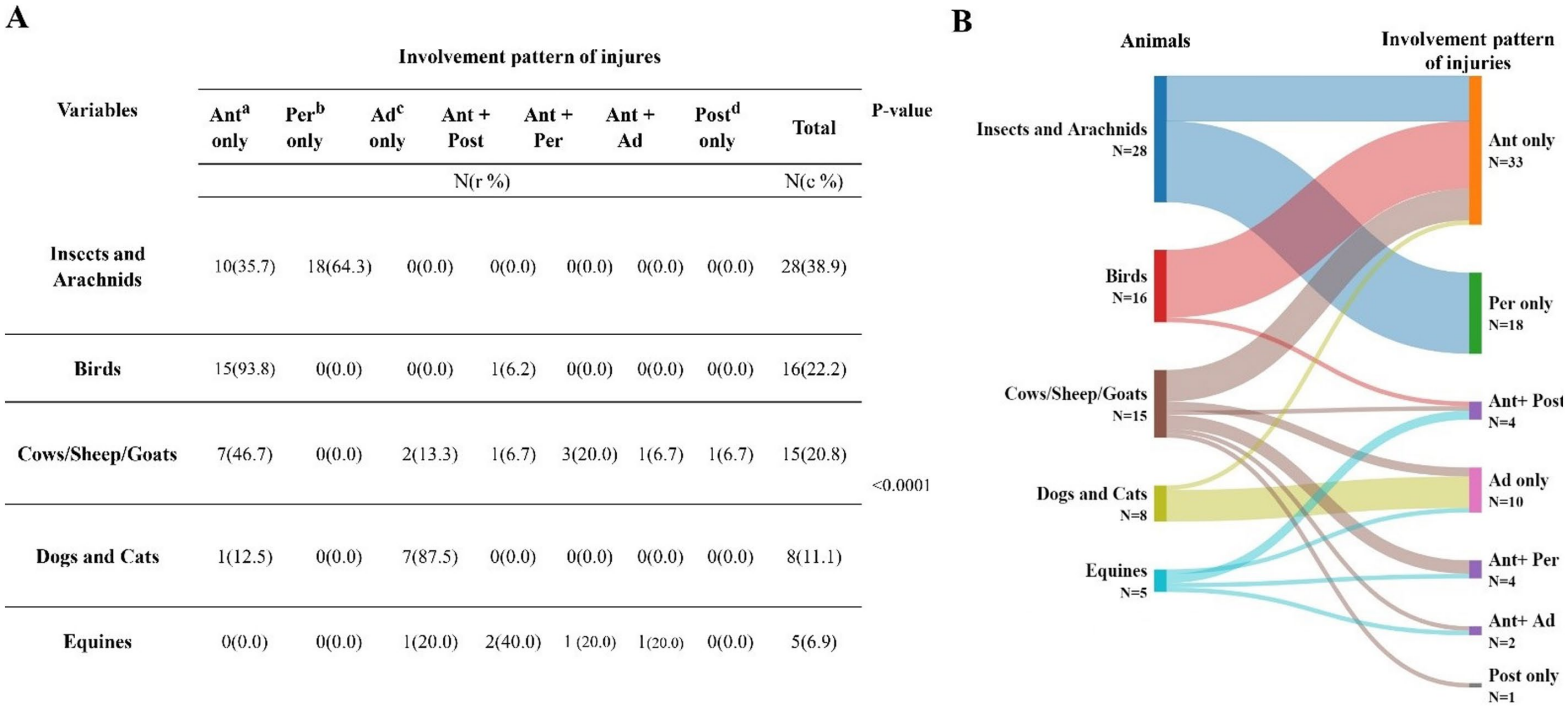
**(A)** The involvement pattern of ocular injuries was significantly associated with the type of animal involved when using Fisher exact tests (*p* < 0.0001). **(B)** Sankey diagram showing the distribution of involvement pattern of injuries according to the animal causing the injury. a. Ant, Anterior; b. Per, Periorbital soft tissue; c. Ad, Adnexal; d. Post, Posterior.

The involvement pattern of ocular injuries was significantly associated with the type of animal involved (*p* < 0.0001; Figure 2). The most common injuries caused by insects and arachnids were periorbital soft tissue injuries (64.3%). Birds were predominantly associated with only anterior segment involvement, occurring in 93.8% of cases. Dogs and cats were significantly related to adnexal injuries, occurring in 87.5% of cases. In the cows, sheep, and goats group, the most frequent injury pattern was the involvement of only the anterior segment (46.7%). However, injuries caused by equines were primarily associated with both anterior and posterior segment involvement (40.0%).

### 3.4 VA and visual impairment severity

Findings on VA and severity of visual impairment at the time of presentation and discharge are summarized in Table 4. The median VA at the time of admission and discharge was 0.15 (IQR 0.7) and 0.04 (IQR 0.15) logMAR, respectively.

**Table 4:** Visual acuity (VA) and visual impairment severity of patients.

Variable		At admission	At discharge
		Median(IQR)	
VA (LogMAR)		0.15(0.7)	0.04(0.15)
Variable		Frequency(%)	
VA (Snellen)	≥ 5/10	46(63.9)	58(80.6)
	5/10 > VA ≥ 1/10	10(13.9)	4(5.6)
	FC	3(4.2)	4(5.6)
	HM/LP	8(11.1)	2(2.8)
	NLP	0(0)	0(0)
	Undetermined	5(6.9)	4(5.6)
Visual impairment severity classification	0 (no impairment)	46(63.9)	58(80.6)
	1 (mild impairment)	4(5.6)	0 (0.0)
	2 (moderate impairment)	6(8.3)	4(5.6)
	3 (severe impairment)	0(0.0)	0(0.0)
	4 (FC)	7(9.7)	5(6.9)
	5 (LP)	4(5.6)	1(1.4)
	6 (NLP)	0(0.0)	0(0.0)
	Undetermined	5(7.0)	4(5.6)

a. FC, Finger counting, b. HM, Hand motion, c. LP, Light perception, d. NLP, No light perception.

The number of patients with no visual impairment increased from 46 cases (63.9%) at the time of presentation to 58 cases (80.6%) at discharge following treatment (Table 4).

Table 5 shows the OTS of patients at the time of admission (median 0.15, IQR 0.7).

**Table 5:** Distribution of ocular trauma scores at the time of admission.

Variable		Median (IQR)
Ocular trauma score		5.0 (1.0)
		Frequency (%)
Ocular trauma score	1	0 (0)
	2	6 (8.3)
	3	6 (8.3)
	4	10 (13.9)
	5	45 (62.5)
	Undetermined	5 (6.9)

There were statistically significant relationships between the type of animal and VA, OTS, and the severity of visual impairment at presentation (*p*-values <0.05; Table 6). Most patients with injuries caused by equines presented with a VA less than 5/10, visual impairment severity categories 1–6, and an OTS less than 5. In contrast, the majority of cases with injuries from other animal groups had a VA of 5/10 or more, an OTS of 5, and a visual impairment severity category of 0 (no visual impairment) at presentation.

**Table 6:** Distribution of VA, OTS, visual impairment severity, and need for surgery according to the type of animal causing injury.

Variables		Insects and arachnids	Birds	Cows/Sheep/Goats	Dogs and cats	Equines
		Frequency (Column %)				
VA (Snellen) at admission	≥ 5/10	21(80.8)	9(69.2)	8(53.3)	7(87.5)	1(20.0)
	5/10 >	5(19.2)	4(30.8)	7(46.7)	1(12.5)	4(80.0)
	*p*-value	0.047				
OTS at admission	1–4	5(19.2)	5(38.5)	7(46.7)	1(12.5)	4(80.0)
	5	21(80.8)	8(61.5)	8(53.3)	7(87.5)	1(20.0)
	*p*-value	0.044				
Visual impairment severity category at admission	0 (no impairment)	21(80.8)	9(69.2)	8(53.3)	7(87.5)	1(20.0)
	1–6 (impairment or blindness)	5(19.2)	4(30.8)	7(46.7)	1(12.5)	4(80.0)
	*p*-value	0.047				
Need for surgery	No	25(89.3)	10(62.5)	10(66.7)	2(25.0)	0(0.0)
	Yes	3(10.7)	6(37.5)	5(33.3)	6(75.0)	5(100)
	*p*-value	<0.001				

Regarding ocular injuries caused by birds, patients attacked by mynah birds had better initial VA (median 0.1 logMAR, IQR 0.1) and final VA (median 0.0 logMAR, IQR 0.0) and higher OTS levels (median 5, IQR 0.5), whereas those injured by roosters or hens had worse initial VA (median 2.6 logMAR, IQR 2.5), final VA (median 1.7 logMAR, IQR 0.8), and lower OTS levels (median 2, IQR 3).

In cases of ocular injuries caused by dogs and cats, the median VA at admission and discharge was 0 logMAR (IQR 0.0) and 0 logMAR (IQR 0.0), respectively.

### 3.5 Management

The management included non-surgical treatments and surgical interventions when necessary.

Twenty-five patients (34.7%) required surgical interventions. The median VA for these patients was 0.39 logMAR (IQR 2.6) at the time of presentation, which improved to 0.15 logMAR (IQR 1.3) at discharge. For patients who underwent only medical therapy, the median VA was 0.09 logMAR (IQR 0.1) at the time of presentation which improved to 0.04 logMAR (IQR 0.0) at discharge.

Details on the distribution of the management strategies are summarized in Table 7.

**Table 7:** Distribution of management strategies performed for animal induced ocular injuries.

Variable		Frequency (%)
Surgical procedures	Foreign body removal	2 (2.8)
	Inferior wall reconstruction	1 (1.4)
	Canthotomy and cantholysis	1 (1.4)
	Anterior chamber washing	1 (1.4)
	Barrier laser	3 (4.2)
	Phacoemulsification	1 (1.4)
	Primary repair	8 (11.1)
	Lid margin repair	1 (1.4)
	Canalicular repair	3 (4.2)
	Suture	7 (9.7)
	Posterior chamber intraocular lens	3 (4.2)
	Pars plana vitrectomy	4 (5.6)
	Total	25 (34.7)
Antibiotic therapy	Cephalexin	24 (33.3)
	Ofloxacin	11 (15.3)
	Fortified vancomycin and ceftazidime	1 (1.4)
	Intravitreal vancomycin and ceftazidime	1 (1.4)
	Chloramphenicol	13 (18.1)
	Total	50 (69.4)
Glucocorticoid therapy	Betamethason	16 (22.2)
	Intravitreal dexamethasone	1 (1.4)
	Prednisolone	7 (9.7)
	Fluorometholone	3 (4.2)
	Total	20 (27.8)
Antihistamine including ketotifen		3 (4.2)
Homatropine		9 (12.5)
Rabie sand Tetanus prophylaxis		8 (11.1)
Contact lens		1 (1.4)
Lubrication (with Hydroxypropyl methylcellulose)		72 (100)

Mean (standard deviation) duration of antibiotic therapy was 10 (3.72) days.

The mean (SD) duration of antibiotic therapy was 10 (3.72) days. The most prevalent antibiotic therapy regimen was oral cephalexin (25 mg/kg/day divided every 6 h for 10 days), followed by topical ofloxacin (0.1% ophthalmic drop 5 mL every 6 h).

In a rare case, the fortified vancomycin (50 mg/mL) and ceftazidime (50 mg/mL) ophthalmic drops were administrated against *Pseudomonas aeruginosa* infection in a patient who presented with a corneal ulceration following a scorpion bite. The initial vision was hand motion perception. After 2 weeks, the infection subsided, but slit lamp examination showed a central corneal scar measuring 3 × 3 mm and his vision remained 1/10 at discharge.

Additionally, we encountered a case of ocular injury caused by an ant. The patient presented with a VA of finger count and a corneal ulcer that did not respond to medical treatment with topical ofloxacin, as the final VA was 1/10.

Topical or systemic corticosteroids were additional medications considered in patients with hyphema. Meanwhile, artificial tears were administered for lubrication and to promote reepithelialization in all patients. The need for surgery in ocular injuries was significantly associated with the animal species (*p* < 0.001; Table 6). Notably, equine-related injuries and injuries caused by dogs and cats required surgery in 100 and 75% of cases, respectively. In our study, out of 15 cases of bee-related ocular injuries, the stinger was observed during the physical examination in two cases, and both underwent foreign body removal using forceps under the slit lamp, followed by antibiotic therapy.

In the birds-related injury group, all the patients with ocular injuries caused by minah birds received non-surgical treatment. However, 75% of patients with injuries caused by roosters and hens required surgical interventions.

### 3.6 Outcomes and complications

**Figure 3:**
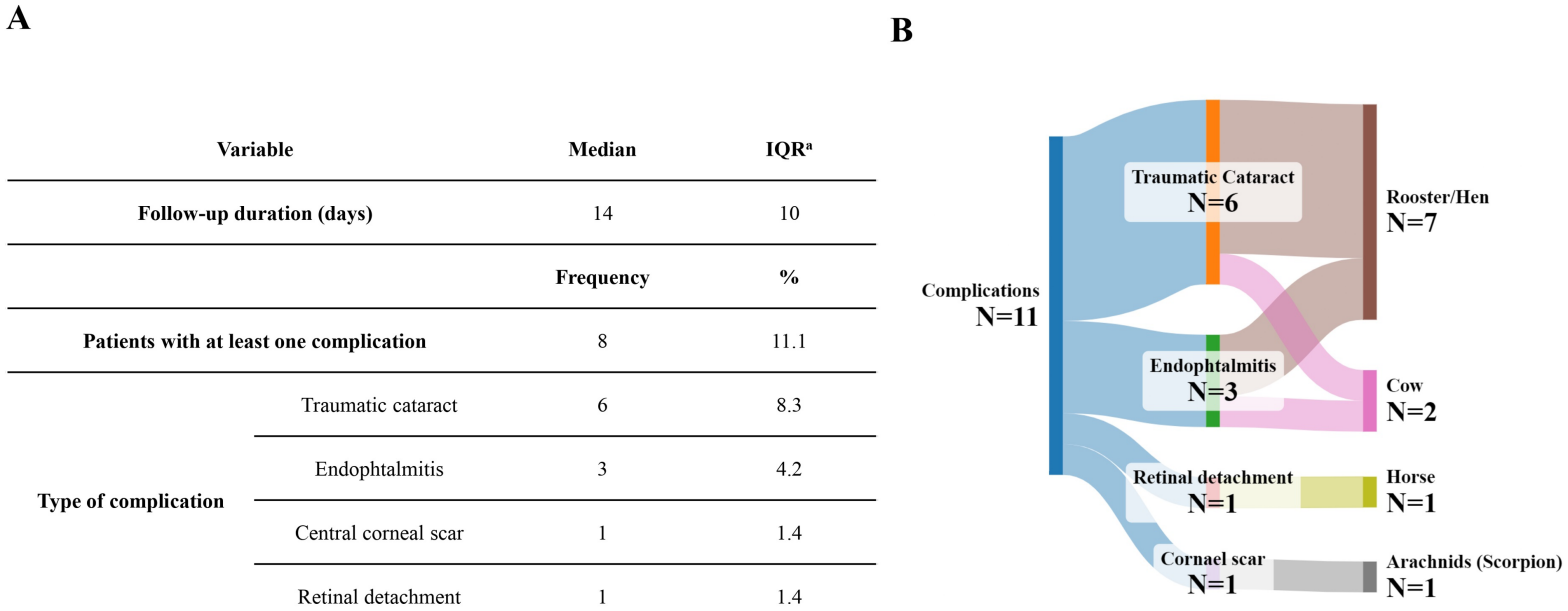
**(A)** The distribution of complications of ocular injuries during the follow-up period. **(B)** Sankey diagram showing the distribution complications according to the animal causing the injury. a. IQR, Interquartile range.

The median follow-up duration was 14 days, with an IQR of 10 days. At the follow-up visit, 11.1% (*N* = 8) of all 72 cases experienced complications (Figure 3). Rooster pecking was the most frequent cause of complications such as endophthalmitis and traumatic cataract. 62.5% (*N* = 5) and 25% (*N* = 2) of patients with injuries caused by hen and rooster pecking developed traumatic cataract and endophthalmitis, respectively.

## 4 Discussion

This prospective study provided the characteristics and outcomes of animal-induced ocular injuries in two tertiary centers over a one-year period.

In our study, insects were the most common cause of injuries. Ocular injuries caused by insects primarily manifested as periorbital soft tissue injuries, followed by involvement of only the anterior segment. The prognosis for insect-related ocular injuries was favorable, as 80.8% of them presented with no visual impairment, and all of them experienced no complications during the follow-up.

Nevertheless, previous studies have reported that insect bite-related ocular injuries have the potential to cause some considerable ocular conditions such as corneal edema, toxic keratopathy, anterior uveitis, toxic scleritis, cataract, optic neuritis, glaucoma, and even endophthalmitis (1). Therefore, maintaining a high level of clinical suspicion and urgent consultation with an ophthalmologist is crucial for managing insect-related ocular injuries.

In our study, the stinger was observed in two cases with bee-related ocular injuries, and foreign body removal was performed for both of them. It is proposed that all venom is released within the first minute following a bee sting, and if a retained stinger is present several minutes after the sting, further venom secretion is unlikely to occur (14). However, removing the foreign body is recommended if it is easily accessible to minimize the potential infection or inflammation as the safest approach (9). Bacterial keratitis following bee stings has also been described in previous reports (14). Therefore, using broad-spectrum topical antibiotics immediately after a bee sting would be prudent as a prophylaxis against potential following infections (14). Previous reports have also recommended the initiation of topical steroid therapy if signs of intraocular inflammation are observed during examination or if there is a decrease in vision (14, 15).

Significantly, we reported a case of corneal ulcer following an ant bite that led to visual impairment. In another study, Amador et al. reported a case of severe keratitis resulting from fire ant stings, which resulted in corneal scarring (16). These cases highlight that ocular injuries caused by ant stings may be severe and should not be underestimated, and if a chemical injury due to the ant venom is suspected, immediate irrigation with regular assessment of the ocular surface pH is recommended (17).

It has been proposed that certain factors, such as iris color, eyeball movements, and corneal sheen, may potentially trigger bird attacks on human eyes (1).

In our study, mynah bird was the most common cause of ocular injury among birds. The lifestyle and demographics of individuals in different regions may influence the types of birds that cause injuries. Mynah birds are popular as pet birds in Iran and Middle Eastern countries, while other bird species may be more common in other parts of the world (18).

In this study, there was a significant association between bird-related injuries and involvement of the anterior segment only.

Tabatabaei et al. (9) described 30 patients with bird attack-related eye injuries. Similarly, in their study, mynah bird was the most common species causing the injuries. They also found that patients attacked by mynah birds had better pretreatment VA than those attacked by hens and roosters, which aligns with our findings.

In our study, none of the cases with ocular injuries caused by minah bird had complications during the follow-up period. Meanwhile, 62.5 and 25% of patients with injuries caused by roosters or hens pecking developed traumatic cataract endophthalmitis, respectively. In this regard, Tobatabaei et al. reported endophthalmitis in 10% of cases with bird pecking-related penetrating globe injuries (18). Therefore, prompt surgical repair and broad-spectrum antibiotic therapy should be considered in bird pecking-related ocular injuries to minimize the risk of developing endophthalmitis (1).

Dog bite injuries are estimated to account for 0.3 to 1.1% of all emergency department visits in the United States annually (19). Approximately 4 to 8% of these injuries involve the periocular area (20). It is estimated that ocular and periocular injuries associated with facial dog bites occur in about 16% of facial dog bite cases and primarily involve the ocular adnexa (7).

In our study, adnexal involvement was the most common pattern of injuries caused by dogs and cats, occurring in 87.5% of cases. These findings agree with those of a previous study by Becerra et al. (21).

Fortunately, severe injuries such as orbital bone fractures and open globe injuries are uncommon in dog bites. However, it is crucial to maintain a high level of suspicion for deep facial, orbital, or globe injuries and consider additional diagnostic measures such as computed tomography when there is clinical suspicion (22).

Infection rates for dog bite injuries have been reported to range from 1 to 17% (6). Dog and cat-inflicted injuries carry a risk of potentially life-threatening conditions such as rabies and tetanus (22, 23). Therefore, prompt medical management, including copious irrigation and conservative debridement, as well as tetanus and rabies prophylaxis, should be considered for involved patients according to their immunization status (22, 23).

In our study, injuries caused by dogs and cats did not lead to a significant reduction in VA, with the median VA remaining 0 logMAR (IQR 0.0) at both admission and discharge. The majority (75%) of patients with injuries caused by dogs and cats required surgical interventions. Fortunately, none of these cases experienced complications during follow-up.

Consistent with prior reports (21, 22, 24–26), in our study, all patients who experienced ocular injuries caused by dogs and cats were bitten by familiar animals. Strategies for preventing dog-induced injuries have shown that educating children on appropriate interactions with dogs, implementing dog ownership licensing, and regulating “dangerous” breeds can reduce these injuries. It is crucial to emphasize that children should never be left unsupervised around dogs to prevent dog bite injuries (3).

In our study, 53.3% of ocular injuries caused by cows, sheep, and goats group did not require surgical interventions, and except for one patient who developed traumatic cataract and endophthalmitis, the others did not develop complications during follow-up.

Nonetheless, cattle-related ocular injuries may have a significant impact, resulting in monocular blindness and the need for eye evisceration due to the posterior extension of globe rupture (27). Ibraim et al. described three male patients with unilateral open-globe injuries and corneoscleral lacerations caused by cow horns. Their initial VA was no light perception in all of them. Two of them required eye evisceration, while the third underwent laceration repair. In their study, none of the patients experienced visual improvement (8).

In the present study, ocular injuries caused by equines were the most severe injuries; notably, all of these injuries required surgical interventions. In this regard, Fleming et al. described 22 patients who experienced neuro-ophthalmic consequences after horse-related head trauma. There were several important neuro-ophthalmic sequelae in their study consisting of orbital fracture, cranial nerve palsy, and traumatic optic neuropathy (11).

We also presented a case of corneal ulceration and vision impairment following a scorpion bite. The patient received Fortified vancomycin and ceftazidime ophthalmic drop, but he experienced central corneal scar as a complication at discharge. We have reported more details about this case in a case report study (28).

Scorpion bites may result in various ocular manifestations, including transient blindness, bilateral optic neuropathy, cerebral blindness, and even vision loss (4). In a similar case, Hamid et al. reported periorbital edema and sudden vision loss in a patient with a scorpion bite on her right eyebrow. Fundus photography showed evidence of occlusion in the superior branch of the retinal vein and retinal hemorrhage caused by the scorpion venom-induced coagulative disorder affecting ocular vasculature. They initiated intravitreal bevacizumab for early management of the branch retinal vein occlusion, and the patient experienced vision recovery 2 weeks after the injection (4).

Untreated ocular injuries can lead to sight-threatening complications, making an immediate response and prompt medical intervention crucial (29, 30). Preventing visual impairments from ocular injuries requires injury prevention, early presentation by the victim, accurate examination, and prompt referral to an ophthalmologist (29).

Healthcare providers at the primary level should stabilize the patient, accurately assess both eyes, and initiate appropriate medical interventions (31). It is important not to manipulate the injured eye, especially in cases of rupture or perforating injuries (31).

Pulling out protruding foreign bodies and using traditional eye medicines should be avoided (31). Instead, a protective shield (not an eye pad) should be placed over the eye without applying pressure, and the patient should be referred to an ophthalmologist without delay to reduce the burden of ocular injuries (29, 31). Additionally, appropriate antibiotic therapy, along with immunoprophylaxis if indicated, should be considered to minimize the risk of potential infectious complications (29, 31).

This study has several limitations. Firstly, it was conducted at two tertiary hospitals in Iran, which might limit its ability to represent injuries caused by animals in other regions and countries with different animal species. Secondly, due to the rarity of these injuries, the number of included cases was limited, which may affect the generalizability of the results. Another limitation is the relatively short and diverse follow-up durations, which could potentially underestimate the long-term outcomes of injuries.

Nonetheless, this study provides valuable information regarding animal-induced ocular injuries, which may improve public awareness and help healthcare providers apply appropriate management strategies. Educating parents and caregivers about the ocular risks of animal interactions may help prevent these injuries and lead to early presentation and prompt referral to an ophthalmologist, resulting in timely diagnosis and minimizing visual loss.

Future studies with larger sample sizes, more extended follow-up periods, and prospective designs are needed to obtain a more accurate assessment of the prognosis of animal-induced ocular injuries.

## Data Availability

The raw data supporting the conclusions of this article will be made available by the authors, without undue reservation.
